# Higher prevalence of splenic artery aneurysms in hereditary hemorrhagic telangiectasia: Vascular implications and risk factors

**DOI:** 10.1371/journal.pone.0226681

**Published:** 2020-01-23

**Authors:** Jacques Sellier, Carma Karam, Alain Beauchet, Axel Dallongeville, Stephen Binsse, Sandra Blivet, Isabelle Bourgault-Villada, Philippe Charron, Thierry Chinet, Mélanie Eyries, Carole Fagnou, Jérome Lesniak, Gilles Lesur, Jérome Lucas, Agnès Nicod-Tran, Augustin Ozanne, Aurélien Palmyre, Florent Soubrier, Mostafa El Hajjam, Pascal Lacombe

**Affiliations:** 1 Ambroise Paré Hospital, AP-HP, Boulogne-Billancourt, University of Versailles-Saint Quentin en Yvelines, France; 2 Pitié-Salpêtrière Hospital, Department of Genetics, AP-HP, Paris, France; Odense University Hospital, DENMARK

## Abstract

**Background:**

Splenic artery aneurysm (SAA) is a rare but potentially fatal condition. Rupture results in 25% mortality up to 75% in pregnant women with 95% fetal mortality. Brief reports suggest an increased risk of developing SAA in patients with HHT.

**Methods:**

We analyzed enhanced multidetector CT data in 186 HHT patients matched (gender and ± 5 year old) with 186 controls. We screened for SAA and recorded diameter of splenic and hepatic arteries and hepatic, pancreatic and splenic parenchymal involvements. We determined by univariate and multivariate analysis, the relationship with age, sex, genetic status, cardiovascular risk factors (CVRF) and visceral involvement.

**Results:**

SAA concerned 24.7% of HHT patients and 5.4% of controls, p<0.001. Factors associated with increased risk of SAA in HHT were female gender (p = 0.04, OR = 2.12, IC 95% = 1.03–4.50), age (p = 0.0003, OR = 1.04, 95% CI = 1.02–1.06) and pancreatic parenchymal involvement (p = 0.04, OR = 2.13, 95% CI = 1.01–4.49), but not type of mutation, hepatic or splenic parenchymal involvements, splenic size or splenic artery diameter or CVRF.

**Conclusions:**

We found a 4.57 higher rate of SAA in HHT patients without evidence of splenic high output related disease or increased CVRF. These results suggest the presence of a vascular intrinsic involvement. It should lead to screening all HHT patients for SAA. The vasculopathy hypothesis could require a change in management as screening of all systemic arteries and even the aorta and to further research in the field.

## Introduction

Hereditary hemorrhagic telangiectasia (HHT), also known as Rendu-Osler-Weber disease is a rare autosomal dominant genetic disease, with an incidence of 1/8.000 to 1/5.000. It is characterized by the development of muco-cutaneous or visceral telangiectasias and arteriovenous malformations (AVMs). The diagnosis of HHT is established in a proband with three or more of the following clinical features: recurrent and spontaneous nosebleeds; mucocutaneous telangiectasias at fingertips, lips, oral mucosa or tongue; visceral AVMs (gastrointestinal, pulmonary, hepatic, cerebral, or spinal), and first-degree family history of HHT. [1,2]. Genetic diagnosis is based on the identification of a mutation in the transforming growth factor-beta (TGF-β) signaling pathway genes: Endoglin (ENG), activin receptor-like kinase 1 (ACVRL 1), or SMAD4 characterized by the juvenile polyposis syndrome (JPS) usually associated with HHT [2,3]. Mutations are not found in approximately 15–20% of HHT families. Visceral manifestations include hepatic (32 to 78% of patients), pulmonary (15–50%), cerebro-spinal (23%), gastrointestinal (80%) and pancreatic (26%) involvements [2,3]. They are responsible for serious complications, especially bleeding and systemic embolism. The guidelines for the diagnosis of visceral involvement, management and prevention of complications were recently updated[2,3]. Some brief reports suggest that there is an increased risk of developing visceral arterial aneurysms especially splenic artery aneurysms (SAA) in patients with HHT [4,5]. SAAs are either discovered incidentally or in symptomatic cases when they rupture with a 25% mortality increasing to 75% among pregnant women with fetal mortality of 95% resulting from hypovolemia and shock [6].

The aims of this study were to determine the prevalence and to describe the characteristics of SAA in a large cohort of HHT patients compared to a control group, and to identify the factors that contribute to their development.

## Methods

We analyzed retrospectively over an 8-year period (May 2005- July 2013) clinical and radiological data from a prospectively acquired database for consecutive HHT patients compared with control patients. All patients provided verbal informed consent. Parental consent was obtained in minor patients. The study protocol was identical for all patients. Patients with a CT exam in another radiological center were excluded. The study was approved by our local Ethics Committee (Comité de Protection des Personnes-Ile de France) as an observational retrospective analysis.

### Patients

#### Target population

Patients with HHT referred to our pluridisciplinary center for an initial screening or for follow-up were evaluated using a thoracoabdominal enhanced MDCT. Diagnosis was confirmed in all HHT patients based on clinical and/or genetic criteria.

#### Control population

The control group patients were chosen as follows: patients not afflicted by HHT who had an abdominal or thoracoabdominal enhanced MDCT with an exploitable early arterial phase, archived on a dedicated PACS reading system allowing post treatment of the images between 2010 and 2013. We excluded patients with disease that may involve splanchnic vascularization (i.e., portal hypertension, acute pancreatitis or cirrhosis), and with lesions involving the liver, the spleen or the pancreas, as well as patients with symptomatic visceral artery aneurysm. CT scan was indicated for cancer, multiple trauma or atherosclerotic arteriopathy.

### Imaging

We used a multidetector helical CT scanner (Philips Brilliance 40 or Siemens Somatom Definition AS 64). Multiphasic examination was performed in the supine position. Thoracoabdominal evaluation concerned the cervicothoracic junction to the lower margin of the kidneys, with an early arterial phase, starting 35 seconds after intravenous injection of iodinated contrast material. The following parameters were used: section thickness 1 mm, pitch 1.25, increment 1 mm, rotation time 0.5 second, 120 kV, and 150 mAs. For the portal phase starting 80 seconds after injection, an abdominal evaluation was performed, from the hepatic dome to the lower margin of the kidneys, using the following parameters: section thickness 2.5 mm, pitch 2, increment 2 mm, rotation time of 0.5 second, 120 kV, and 200 mAs.

A nonionic contrast material (Iobitridol 300, Xenetix, Guerbet® or Iohexol 300, Omnipaque, GE Healthcare SAS®) was injected into the antecubital vein through a 16–18 gauge needle. We used an automatic injector, with a flow rate of 4–5 mL/sec, and a maximum volume of 140mL.

#### Image evaluation

Acquired data were transferred to a dedicated PACS reading system and reconstruction workstation (Carestream Health Vue PACS®). Multiplanar reconstructions (MPR) and Maximum Intensity Projections (MIP) images were obtained. All exams were read by two experienced senior radiologists, with at least 5 and 20 years of experience with HHT, respectively. A consensual interpretation was obtained in all cases.

### Data collection

The splenic artery aneurysm was defined as a focal dilation in its diameter of > 50% compared to the normal vessel diameter. The vascular analysis consisted in detection, enumeration and sizing of SAA. We measured the maximal transverse diameter of the aneurysmal sac in fusiform aneurysms and the largest diameter of the aneurysmal sac in sacciform aneurysms. In case of multiple SAA, the measurements of the two largest aneurysms were reported.

Three locations of aneurysms were distinguished: intra-parenchymal aneurysm, truncular aneurysm defined as SAA located from the origin of the splenic artery to the first division of the splenic artery and hilar SAA defined as located from the first division of splenic branches to the splenic parenchyma. Multiple locations were defined as the association of two or more of the previously described locations. The diameter of the splenic artery at the junction of the body and the tail of pancreas was measured, as well as the hepatic artery distally to the gastro-duodenal artery. In case of multiple hepatic arteries (middle, left or right hepatic arteries, according to the anatomical hepatic arterial supply), the sum of the diameters of hepatic arteries was calculated.

The vertical long axis of the spleen was measured in a coronal plane. Telangiectasia, were defined as a focal contrast enhancement at the arterial phase, with homogenization at portal phase. Hemangioma were defined as homogeneous or ring enhancement at the arterial phase with portal filling or homogenization at the portal phase; and splenic angiomatosis was defined as multiple hemangiomas or early, diffuse and intense enhancement at arterial phase. The vertical long axis of the liver was measured in a coronal plane. Parenchymal,arterio-portal, arterio-hepatic or porto-hepatic shunts, hypervascular intra-hepatic lesions, including telangiectasia (<10mm), confluent vascular mass (>10mm) or pseudo focal nodular hyperplasia, and haemangioma were monitored as previously described [7].

According to the description by Lacout et al [8], we defined pancreatic involvement as the presence of telangiectasia, identified as focal lesions detected at the arterial phase, with partial or complete homogenization at the portal phase, and AVMs.

We reported the occurrence of pulmonary AVMs, characterized by an enlarged feeding pulmonary artery directly connected to a draining vein through an aneurysmal sac or a serpiginous network. The pulmonary involvement was assessed on thin slices images (0.5mm), with a pulmonary window setting.

Cardiovascular (CV) risk factors were hypertension, smoking, diabetes, dyslipidemia and family history of CV disease.

### Statistical analysis

Statistical analyses were performed using R software version 3.0.2 (http://www.r-project.org).

Quantitative data are expressed as mean ± standard deviation, qualitative data as frequency and percent. Comparisons of means were performed using the Student's t-test, and comparisons of frequencies using the Chi square test and the Fisher exact test. A multiple logistic regression was carried out to identify risk factors of SAA. A p value ≤ 0.05 was considered significant. In both groups, data were adjusted according to gender and ± 5-year old age.

## Results

**Fig 1:**
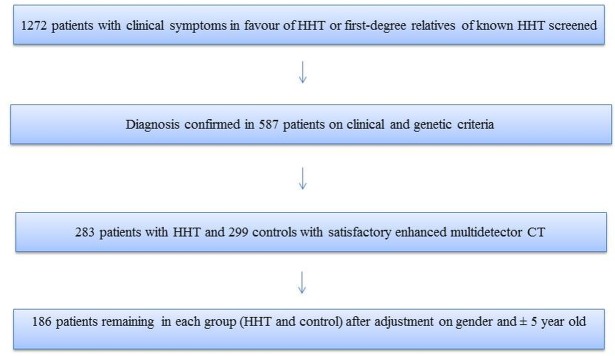
Flow chart.

Fig 1 illustrates the selection of HHT and control group patients (flow chart). Initially, 283 HHT patients were included and 299 in the control group. Both groups were not homogeneous. There were more women in HHT group (59.7% vs 42.5% in the control group, p<0.0001). Moreover, HHT population was statistically younger (41.8 ± 18.4 years vs 59.3 ± 20.8 years in the control group, p<0.0001). After adjustment according to gender and age, we obtained two homogeneous groups of 186 patients [gender: p = 1, mean age 46.4 ± 18.5 years (HHT); 49.1 ± 13.3 years (controls), p = 0.86] (S1 Table). The main characteristics of population with SAA are summarized in Table 1. SAA were significantly more frequent in HHT than in controls (24.7% vs 5.4%, p < 0.0001). The mean age of HHT patients with SAA was 57.1 ±14 years vs 69.7 ±11.2 in controls (p = 0.007). In the HHT group, 32.6% of women had SAA vs 17.5% of men (p = 0.017), and SAA were more often described in women than in men (63% vs 37%). SAA occurred less often in controls, exclusively in women (11.2%).

**Table 1:** Characteristics of all patients with splenic artery aneurysm (SAA).

	Total HHT n = 186	Total controls n = 186	SAA HHT n = 46	SAA control P value n = 10
Age (years) ± SD	49.1 ± 18.3	46.4 ± 18.5	57.1 ± 14	69.7 ± 11.2 0.01
Women, %	89 (47.8%)	89 (47.8%)	29 (63%)	10 (100%) 0.02
Men, %	97 (52.2%)	97 (52.2%)	17 (37%)	0 (0%) 0.02
Women with SAA, %	29/89 (32.6%)	10/89 (11.2%)		
Men with SAA, %	36/97 (37%)	0/97 (0%)		

SD = Standard Deviation

Tables 2 and 3 describe SAA location in HHT and control patients. The total number of SAA was 8.7 times higher in HHT than in controls (113 vs 13 respectively), but the number of SAA per patient (1.71 ± 1.26 in HHT vs 1.2 ± 0.42 in controls) was not statistically different (p = 0.16). The size of the largest SAA was 17 mm and the main aneurysm size (8.62 ± 3.37 mm in HHT vs 6.70 ± 2.80 mm in controls) was not different between groups (p = 0.09). Most HHT patients had SAA located only at the hilum (25/46), five had truncular location, two had intraparenchymal location and fourteen had multiple locations, mainly hilar and truncular (9 patients). In the control group, SAA were mainly hilar (9/10) and intra-parenchymal (1/10).

**Table 2:** Characteristics of the splenic artery aneurysms (SAA).

	HHT patients n = 46	Control Group n = 10	P Value
Total number of SAA	113	13	
SAA per patient ± SD, (range)	1.71± 1.26 (1–8)	1.20±0.42 (1–2)	0.16
Size (biggest, mm) ± SD, (range)	8.62 ±3.37 (2.5–17)	6.70±2.8 (2–13)	0.09
Hilar location	25	9	
Truncular location	5	0	
Intra parenchymal location	2	1	
Multiple locations	14	0	

SD = Standard Deviation

**Table 3:** Splenic and hepatic artery diameter in both groups, in case of the presence or absence of HHT in SAA patients, in case of the presence of absence of SAA in HHT patients.

	N	Hepatic artery diameter (mm)±SD (range)	P Value	Splenic artery diameter(mm)±SD (range)	P Value
In both groups					
HHT patients	186	8.31±3.38(3.0–24.0)		5.48±1.27(2.8–9.0)	
Control group	186	5.92±1.51(3.0–11.0)	< 0.0001	5.45±1.04(3.3–10.5)	0.77
In patients with SAA					
HHT patients	46	9.27±3.45(4.6–20.8)		5.76±1.47(3.5–9.0)	
Control group	10	5.31±0.96(4.4–7.7)	< 0.0001	5.75±2.07(4.2–10.5)	0.98
In HHT patients					
With SAA	46	9.27±3.45(4.6–20.8)		5.76±1.47(3.5–9.0)	
Without SAA	140	8.0±3.31(3.0–24.0)	0.03	5.39±1.18(2.8–9.0)	0.08

SAA = splenic artery aneurysm

SD = Standard Deviation

HHT = Hereditary Hemorrhagic Telangiectasia

The diameter of the splenic artery was not statistically different between groups (5.48 ± 1.27 mm in HHT vs 5.45 ± 1.04 mm in controls, p = 0.77). It was similar in case of SAA in HHT patients and controls (5.76 ± 1.47 mm vs 5.75 ± 2.07 mm; p = 0.98). Moreover, it was not different between HHT patients with or without SAA (5.76 ± 1.47 mm vs 5.39 ± 1.18 mm; p = 0.08). Conversely, the diameter of the hepatic artery was statistically larger in case of SAA (9.27 ± 3.45 mm vs 5.31 ± 0.96 mm, p < 0.001).

**Fig 2:**
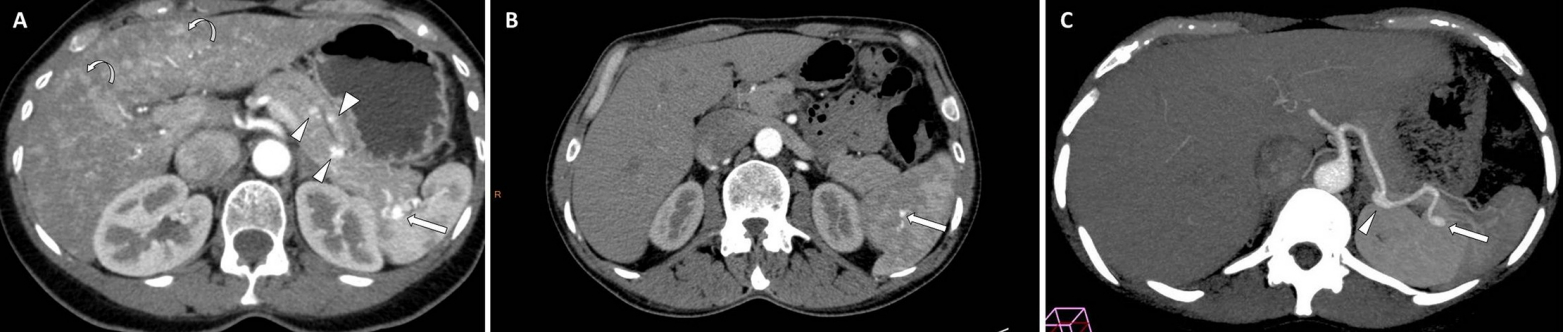
**A.** SAA in a 52 year-old man with HHT and parenchymal pancreatic involvement. Enhanced computed tomography in axial view show hilar splenic artery aneurysm (arrow) associated with multiple pancreatic telangiectasia (arrowheads). Note the hepatic involvement characterized by heterogeneous enhancement due to multiple hepatic telangiectasia (curved arrows). **B.** SAA in a 54 year-old man with HHT and a splenic intraparenchymal aneurysm. Enhanced computed tomography in axial view show intraparenchymal splenic artery aneurysm (arrow). **C.** SAA in a 44 year old woman with multiple locations of SAA (truncular and hilar). Enhanced computed tomography in axial view and maximal intensity projection (MIP) reformation showing an hilar (arrow) and a truncular splenic artery aneurysm (arrowhead).

Fig 2 illustrates the association between a hilar SAA and multiple pancreatic telangiectasia. Pancreatic involvement was present in 64/186 HHT patients and in none of the patients in the control group (p <0.0001). In HHT patients, SAA were significantly more frequent in case of pancreatic involvement (39.1% vs 17.2%, p = 0.001). However, there was no anatomic contiguity between the pancreatic telangiectasia and the SAA.

**Fig 3:**
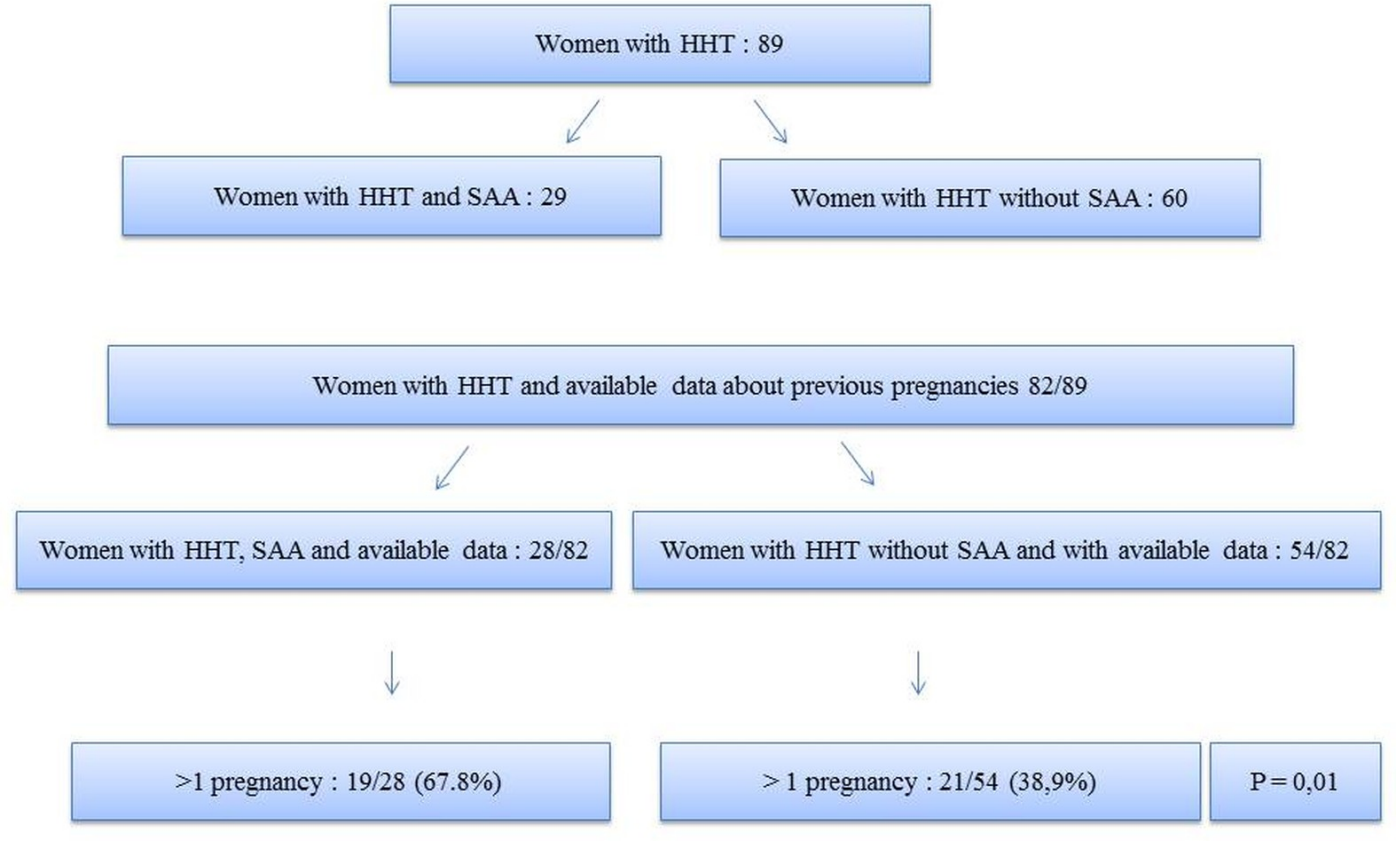
Women with HHT, previous and multiple pregnancies.

Tables 4 and 5 show CV risk factors and pregnancy in HHT patients and controls, with or without SAA. Unlike other characteristics, data concerning CV risk factors and pregnancy were obtained in 161 HHT patients and 77 controls. There was no statistical difference in terms of hypertension, smokers and dyslipidemia between HHT and controls. Diabetes and familial history of CVD were found to be significantly lower in HHT compared to controls. The CV risk factors were not statistically different in patients with or without SAA in both groups. We found significantly more SAA in women with multiple pregnancies in the HHT population (Fig 3, 67.8% vs 38.9% p = 0.01).

**Table 4:** Cardiovascular risk factors in HHT patients and controls.

	HHT patients n = 161	Control Group n = 77	P Value
Mean age (years)	45.6 ± 18	47.3 ± 17.1	0.13
Hypertension (%)	27 (16.8%)	20 (26%)	0.13
Smokers (%)	58 (36%)	36 (46.7%)	0.15
Diabetes (%)	9 (5.6%)	12 (15.6%)	0.02
Dyslipidemia (%)	18 (11.2%)	13 (16.9%)	0.31
History of CVD (%)	30 (18.6%)	30 (39%)	0.001

CVD = Cardiovascular Disease

HHT = Hereditary Hemorrhagic Telangiectasia

**Table 5:** Cardiovascular risk factors and pregnancy in HHT patients and controls with or without SAA.

	HHT patients		P value	Control patients		
	with SAA	without SAA		with SAA	without SAA	P value
	n = 42	n = 119		n = 2	n = 75	
Hypertension	9 (21.4%)	18 (15.1%)	0.35	1 (50%)	12 (16%)	0.31
							Smokers	15 (35.7%)	43 (36.1%)	0.96	0 (0%)	34 (45.3%)	0.50
Diabetes	4 (9.5%)	5 (4.2%)	0.24	1 (50%)	7 (9.3%)	0.20							
Dyslipidemia	7 (16.7%)	11 (9.2%)	0.25	0 (0%)	13 (17.3%)	1
History of CVD	11 (26.2%)	19 (15.9%)	0.12	0 (0%)	27 (36%)	0.54
Previous pregnancy in women	21/28 (75%)	31/54 (57.4%)	0.12	2/2 (100%)	31/41 (41.3%)	1
Pregnancies > 1	19/28 (67.8%)	21/54 (38.9%)	0.01	2/2 (100%)	25/41 (33.3%)	0.52

CV = Cardiovascular

CVD = Cardiovascular Disease

SAA = Splenic Artery Aneurysm

HHT = Hereditary Hemorrhagic Telangiectasia

The factors associated with SAA in HHT patients are summarized in Table 6. The factors that significantly increased the risk of developing SAA were female gender (p = 0.04, OR = 2.12, IC 95% = 1.03–4.50), age (p = 0.0003, OR = 1.04, 95% CI = 1.02–1.06) and pancreatic parenchymal involvement (p = 0.04, OR = 2.13, 95% CI = 1.01–4.49). The type of mutation, the pulmonary, hepatic or splenic parenchymal involvements and the splenic and hepatic sizes were not correlated to an increased risk of SAA.

**Table 6:** Factors associated with SAA in HHT patients.

	HHT patients with SAA n = 46	HHT patients without SAA n = 140	Univariate	Multivariate	OR (95% CI)
**Gender (female)**	29 (63%)	60 (43%)	0.017	0.04	2.12 (1.03–4.50)
**Age (years)**	57 ± 14	43 ± 18	<0.0001	0.0003	1.04 (1.02–1.06)
**Type of mutation A/E**	27A/17E	61A/62E	0.18		
**Pulmonary Involvement**	31 (37%)	82 (58%)	0.29		
**Hepatic Involvement**	33 (72%)	97 (69%)	0.75		
**Splenic Involvement**	9 (19%)	17 (12%)	0.21		
**Pancreatic Involvement**	25 (54%)	39 (28%)	0.001	0.04	2.13 (1.01–4.49)
Maximal Size of the Spleen (mm)*	10.5 ± 1.4	10.5 ± 1.7	0.99		
Maximal Size of the Liver (mm)*	16.5 ± 1.7	16.7± 2.1	0.41		

OR = Odd Ratio, CI = Confidence Interval

A = ACVRL1, E = Endoglin

SAA = Splenic Artery Aneurysm, HHT = Hereditary Hemorrhagic Telangiectasia

*Organ size measured by the vertical long axis in a coronal plane

## Discussion

This is the first study to address the high prevalence of SAA in a large cohort of HHT patients. We found SAA in 24.7% of HHT patients and 5.4% in the control group (p < 0.0001). HHT affects visceral organs, especially the lung, brain, spinal cord and liver, and involve visceral arteries (gastrointestinal, pulmonary, hepatic, cerebral, or spinal). Blood flow increase can cause arterial aneurysms in these territories, mainly in the hepatic artery.

In the general population, SAA were first described in 1770 by Beaussier [9]. They represent the most frequent splanchnic artery aneurysms (60%)[10]. The prevalence rate varies from 0.098% to 10.4% in autopsies [11,12]. Female are four times more often affected than male [10].

Furthermore, SAA have been poorly addressed in HHT, mainly through case reports in women [13–15]. In 2004, Delvi et al. detailed the surgical care in a 28 year-old HHT woman diagnosed with a SAA treated by excision of the splenic aneurysm [16].

Recently, Moulinet et al. found a higher frequency of visceral aneurysms in HHT patients (8 / 91, 8.8%) than in controls (2 / 226, 0.92%). Four of these patients presented a SAA[4].

In non HHT patients, SAA are associated with trauma, hormonal and local hemodynamic events during pregnancy, portal hypertension, arterial degeneration (medial fibrodysplasia), polyarteritis nodosa, alpha-1-antitrypsin deficiency and atherosclerosis [17–19].

In our study, age, gender and pancreatic involvement significantly increased the risk of developing SAA in HHT patients.

There was a significant difference in age between HHT patients and controls, who were older (mean age 57.1 years vs 69.7 years, respectively, p = 0.007). This is possibly related to arteriosclerosis in the general population, but not in HHT patients, in which other factors may interfere.

Yet, age is associated with the progression of visceral involvement in HHT. Telangiectasias increase with age and gastrointestinal tract and liver involvements are classically seen in older HHT patients [19–21]. Hepatic AVMs worsen with age, and lead to splanchnic blood flow increase and therefore high output cardiac failure in HHT patients [22].

In the control group, SAA were exclusively present in women. This predominance in the general population is supported by most studies in the literature. The influence of parity and pregnancy was strongly suggested by several authors [17–19, 23–26]. During pregnancy, weakness in the arterial wall influenced by hormones (estrogen and progesterone), increase of blood volume and cardiac output, and portal hypertension contribute to the development of SAA [19].

In HHT patients, 63% of SAA concerned women while 37% were encountered in men (HHT vs control 37% vs 0%, p< 0.001). Our results showed that SAA also affect men, whereas this pathology seems exclusively feminine in the control group in our study. Moulinet et al. observed SAA in four HHT patients of whom one was a man (25%) [4].

In our study, pancreatic parenchymal involvement was significantly associated with the risk of developing SAA (Table 6). Similarly, Lacout et al observed a trend toward larger diameters of the splenic artery in patients with pancreatic AVMs [8]. However, since there was no anatomic contiguity between the pancreatic telangiectasia and the SAA, the splenic aneurysm is not to be considered as the resultant of pancreatic telangiectasia.

In our study, splenic artery diameter was similar in HHT patients with SAA and without SAA (Table 3). Furthermore, there was no association between SAA and splenic parenchymal involvement or splenic size. Finally, liver and splenic parenchymal involvements were not significantly associated with the risk of developing SAA.

**Fig 4:**
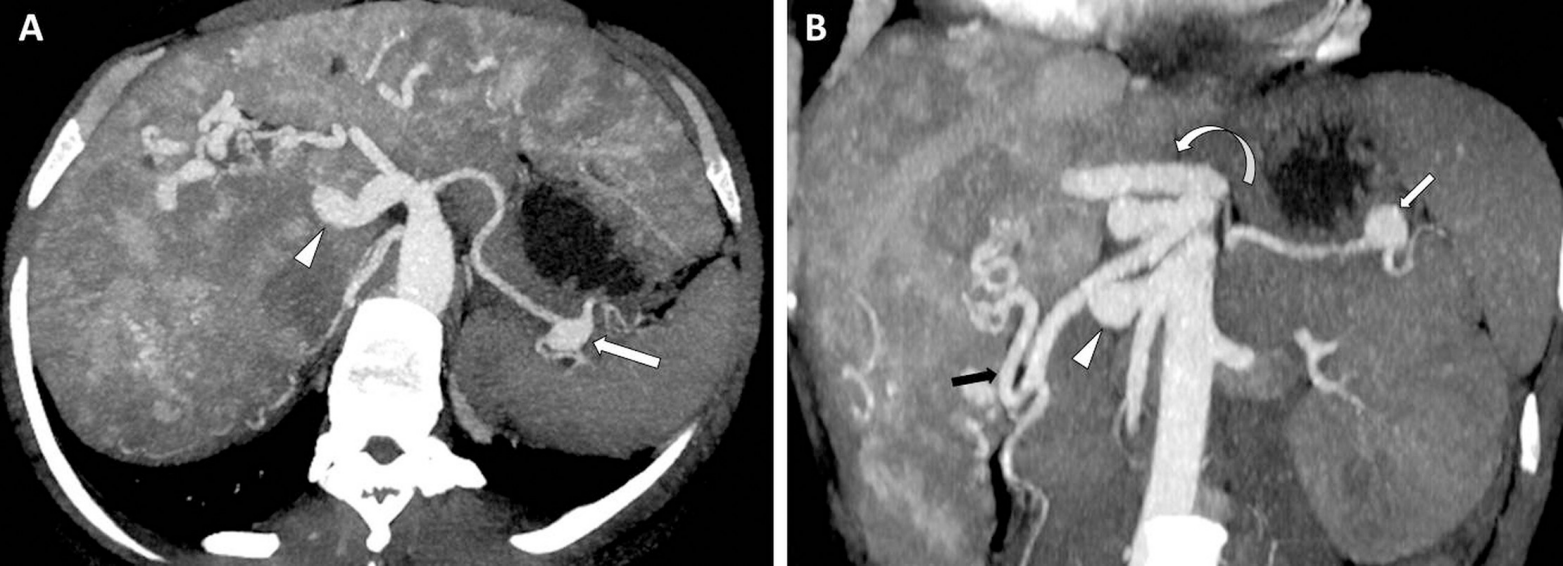
SAA in a 50 year-old woman with HHT and severe liver involvement. Enhanced computed tomography in oblique axial (**A**) and oblique coronal (**B**) Maximal Intensity Projections (12mm thickness). **A.** a 12x17mm fusiform hilar SAA is supplied by a 4mm splenic artery (arrow). The severe liver involvement is characterized by enlargement of arteries supplying the liver (right hepatic artery, 15.9 mm: arrowhead), by presence of arterio-portal and arterio-hepatic shunts and by hepatomegaly with heterogeneous enhancement. **B.** Three enlarged hepatic arteries supply the liver: A 4.5mm middle hepatic artery originating from the coeliac trunk (thin arrow). A 8mm left hepatic artery originating from the left gastric artery (curved arrow). A 12 mm right artery originating from the superior mesenteric artery (arrow). The increased hepatic blood flow contrasts with the splenic blood flow, which seems to be decreased by a probable steal phenomenon. Then, the SAA (star) has developed on a systemic artery, in spite of a decreased blood flow induced by the severe hepatic involvement. This reinforces the hypothesis of a vasculopathy at the origin of SAA.

We found a 4.57 higher rate of SAA in HHT patients compared to the control group without evidence of a splenic high output related disease (normal spleen in size, the absence of splenic parenchymal involvement, similar splenic artery diameter between HHT patients and control group). Thereby, the hypothesis of a non-visceral intrinsic vascular involvement or of a vasculopathy should be raised. Hence, HHT patients could develop a “vasculopathy”, independently of visceral parenchymal involvement and of high splanchnic blood flow (Fig 4).

Some patients and families with JPS and SMAD4 mutation exhibit a phenotype of HHT with epistaxis, telangiectasia and visceral AVM, particularly hepatic and pulmonary, without a genetic HHT mutation. These patients are known to have a predilection for aortic root aneurysms identical to those found in Marfan's disease [27]. Therefore, there may be an overlap between HHT disease and intrinsic pathology of the aorta.

Several authors hypothesized a central role of TGF-β signaling pathway components known to be involved in HHT gene mutations (ENG, ACVRL 1) and implicated in the pathophysiology of atherosclerosis, and other hereditary diseases like primary pulmonary hypertension and aortic aneurysm syndromes (Marfan, type IV Ehlers-Danlos and Loeys-Dietz syndromes) [20,28]. Indeed, TGF-β signaling pathway classically induces the deposit of extracellular matrix (ECM) within the vascular wall, while repressing ECM degradation. These disturbances may be detrimental to normal vascular function and architecture, and endothelial cell response [20,28].

In our study, there was no association between the type of mutation (ENG, ACVRL1), pulmonary involvement and the presence of SAA in HHT patients. If the development of SAA was mainly related to a splanchnic hyper blood flow factor, we would expect to find a predominance of ACVRL1 vs ENG mutation, since pulmonary AVMs are more common in individuals with ENG pathogenic variants, and hepatic AVMs are more common in individuals with ACVRL1 pathogenic variants [29].

The absence of genetic predominance is an additional element in favor of the existence of an intrinsic systemic arterial involvement.

However, our study has some limitations. Only some patients screened for HHT had a CT scan in our center. Only complete scans with arterial time and portal time were included, and only CT scans of good imaging quality were selected. This could represent a selection bias.

We adjusted our data on gender and age, to obtain two homogeneous and comparable groups. This led to a reduction in the size of the two groups, and therefore a loss of power. Nevertheless, the number of patients remained high (186 in each arm) and we were able to demonstrate a significant increase in SAA in HHT patients. We did not collect prospectively arteriosclerosis risk factors (except age and gender) and analyze the parity or the number of pregnancies in women prospectively, but we obtained the information retrospectively and therefore, some data are missing, particularly in the control group.

In conclusion, this is the first study to demonstrate a higher prevalence of SAA in a large cohort of HHT patients, comparatively to the control population. These results suggest the presence of a vascular intrinsic involvement. These data should lead to screening all HHT patients for SAA, since they require therapeutic management (embolization or surgery) in case of a symptomatic or a large aneurysm [17–19]. A particular attention should be paid to women of childbearing age, since pregnancy could be a risk factor for aneurysm rupture, and could imply a preventive treatment. SAA are found more often in HHT women with multiple pregnancies. The vasculopathy hypothesis could require a change in management as screening of all systemic arteries and even the aorta. It should also lead to further research in the field.

## Supporting information

S1 Table (XLS)

## Abbreviations

HHT: hereditary hemorrhagic telangiectasia
AVMs: arteriovenous malformations
TGF-β: transforming growth factor-beta
ENG: endoglin
ACVRL 1: activin receptor-like kinase 1
JPS: juvenile polyposis syndrome
SAA: splenic artery aneurysms
MDCT: enhanced multidetector CT
MPR: multiplanar reconstructions
MIP: maximum intensity projections
ECM: extracellular matrix
CV: Cardiovascular
